# A Modified Palmar Approach With Tendon Splitting for Distal Phalanx Enchondromas of the Thumb: A Report of Two Cases

**DOI:** 10.1016/j.jhsg.2025.01.001

**Published:** 2025-01-27

**Authors:** Yukihiro Kokubu, Keisuke Hirose, Toshinori Kurashige, Yoshiaki Ando, Kiyoma Marusugi, Hiroshi Kawaguchi

**Affiliations:** ∗ Department of Orthopedic Surgery, Nadogaya Hospital, Chiba, Japan

**Keywords:** Distal phalanx, Enchondroma, Minimally invasive surgery, Palmar approach, Thumb

## Abstract

Distal phalanx enchondromas of the thumb are rare and pose unique challenges for surgical management because of the thumb’s critical role in hand function. Traditional dorsal and lateral approaches risk damaging extensor tendons, the nail matrix, limiting interphalangeal mobility, or compromising pinch function. This report presents two cases of thumb distal phalanx enchondromas successfully treated using a modified palmar approach with Bruner’s incision followed by splitting the insertion of the flexor pollicis longus tendon. Both patients achieved complete curettage, bone regeneration, and full preservation of thumb function at the 1-year follow-up. The incision design avoided high-pressure zones of the pulp, reducing postoperative complications while maintaining functionality. Fluoroscopic guidance facilitated effective curettage through the flexor pollicis longus tendon split without extensive exposure. These findings underscore the tendon splitting palmar approach as a viable option in selected cases, highlighting the importance of individualized surgical strategies to optimize outcomes for distal thumb enchondromas.

Enchondromas are benign cartilaginous tumors commonly found in the phalanges of the hand. Although some are incidentally discovered during radiographic imaging for unrelated conditions, most present with symptoms such as pain or pathological fractures. Enchondromas predominantly affect the proximal and middle phalanges, with those occurring in the distal phalanx being rare, and even more so in the thumb.^1^^,^^2^ This report describes two cases of distal phalanx enchondromas in the thumb treated surgically.

Surgical approaches for enchondromas are well documented, with dorsal and lateral incisions being commonly employed for lesion curettage, with or without bone grafting. A review of 104 cases of enchondromas in the phalanges revealed postsurgical losses of joint range of motion of 3% in the metacarpophalangeal joint, 9% in the proximal interphalangeal joint, and 13% in the distal interphalangeal joint.^3^ These findings underscore the delicate nature of distal interphalangeal joint function and the importance of carefully selecting surgical approaches for enchondromas in the distal phalanx. The dorsal approach carries the risk of damaging the nail matrix and extensor tendons, potentially causing postoperative nail deformities or compromised extension function.^4^ Although the lateral approach reduces the risk of nerve, vessel, and tendon damage, anatomical variations can make adequate exposure and maneuverability challenging. Cases with postoperative pain or limited range of motion have also been reported.^5^ Additionally, in the thumb, a surgical scar between the thumb and index finger may impair lateral pinch function.

The palmar approach, although advantageous in certain clinical scenarios, has been less frequently reported for enchondromas in the distal phalanx. Shimizu et al^6^ described five cases of distal phalanx enchondromas managed via a palmar approach using a longitudinal incision that preserved the pulp 10 mm from the fingertip, effectively avoiding sensory deficits. However, this traditional palmar approach has been associated with painful scars extending to the central pulp, potentially affecting pinching activities.^7^ Although these reports focus on other digits, the thumb’s unique anatomical, and functional importance necessitates careful surgical planning. Given the critical role of the thumb in hand function, a balance must be struck between effective surgical management and preservation of dexterity and strength.

In this report, we introduce a modified palmar approach for two cases of distal phalanx enchondromas of the thumb. We further discuss optimal surgical strategies, focusing on lesion accessibility, surgical exposure, and postoperative outcomes.

## Case Reports

### Case 1

**Figure 1:**
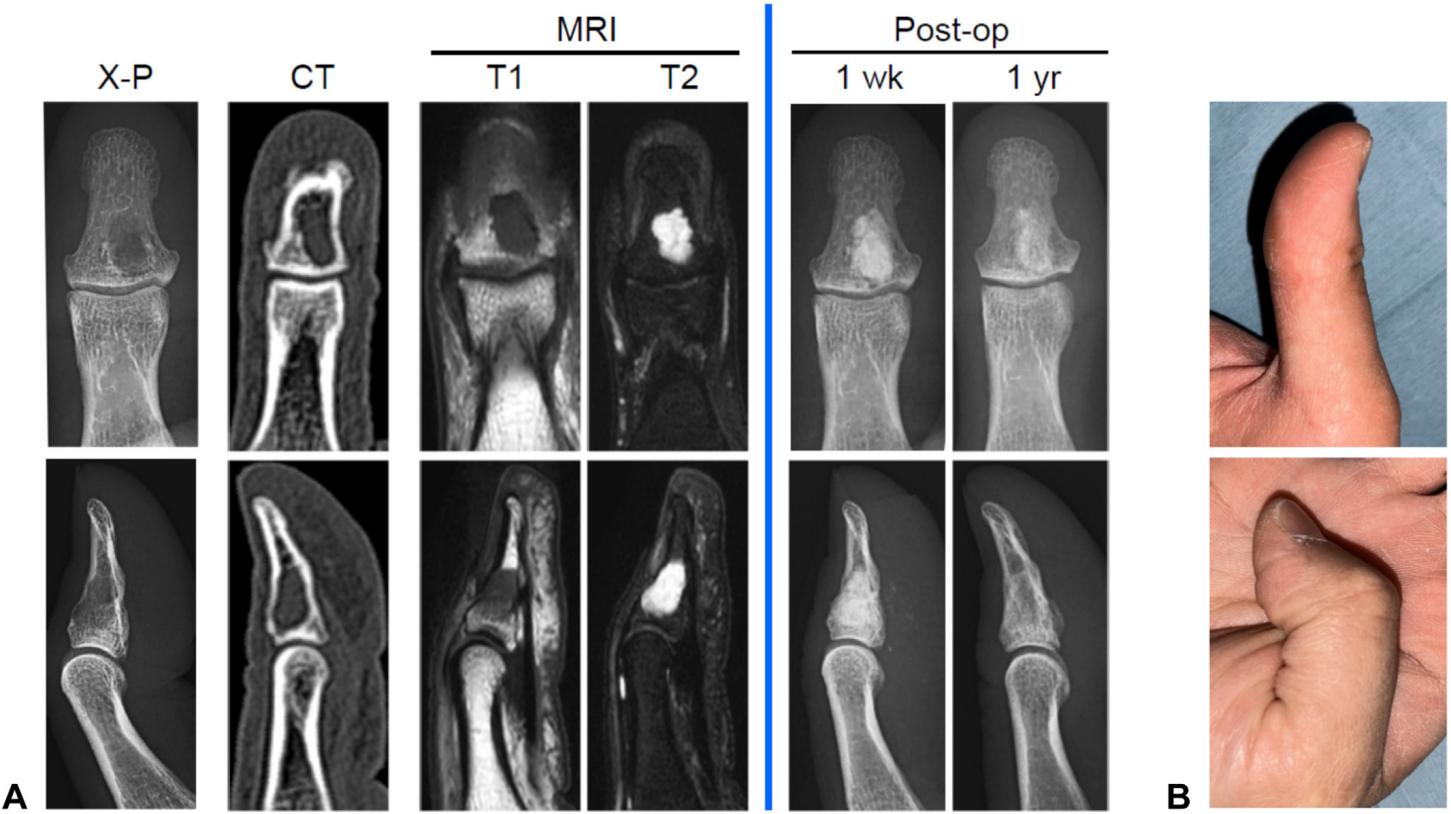
Pre- and postoperative findings for case 1. **A** Left of the blue line: Preoperative radiographs, computed tomography, and magnetic resonance imaging (T1- and T2-weighted images), showing frontal (upper row), and sagittal (lower row) views. Right of the blue line: Postoperative radiographs at 1 week and 1 year, with frontal (upper row), and lateral (lower row) views. **B** Thumb range of motion at 1 year after surgery: extension (upper) and flexion (lower).

**Figure 2:**
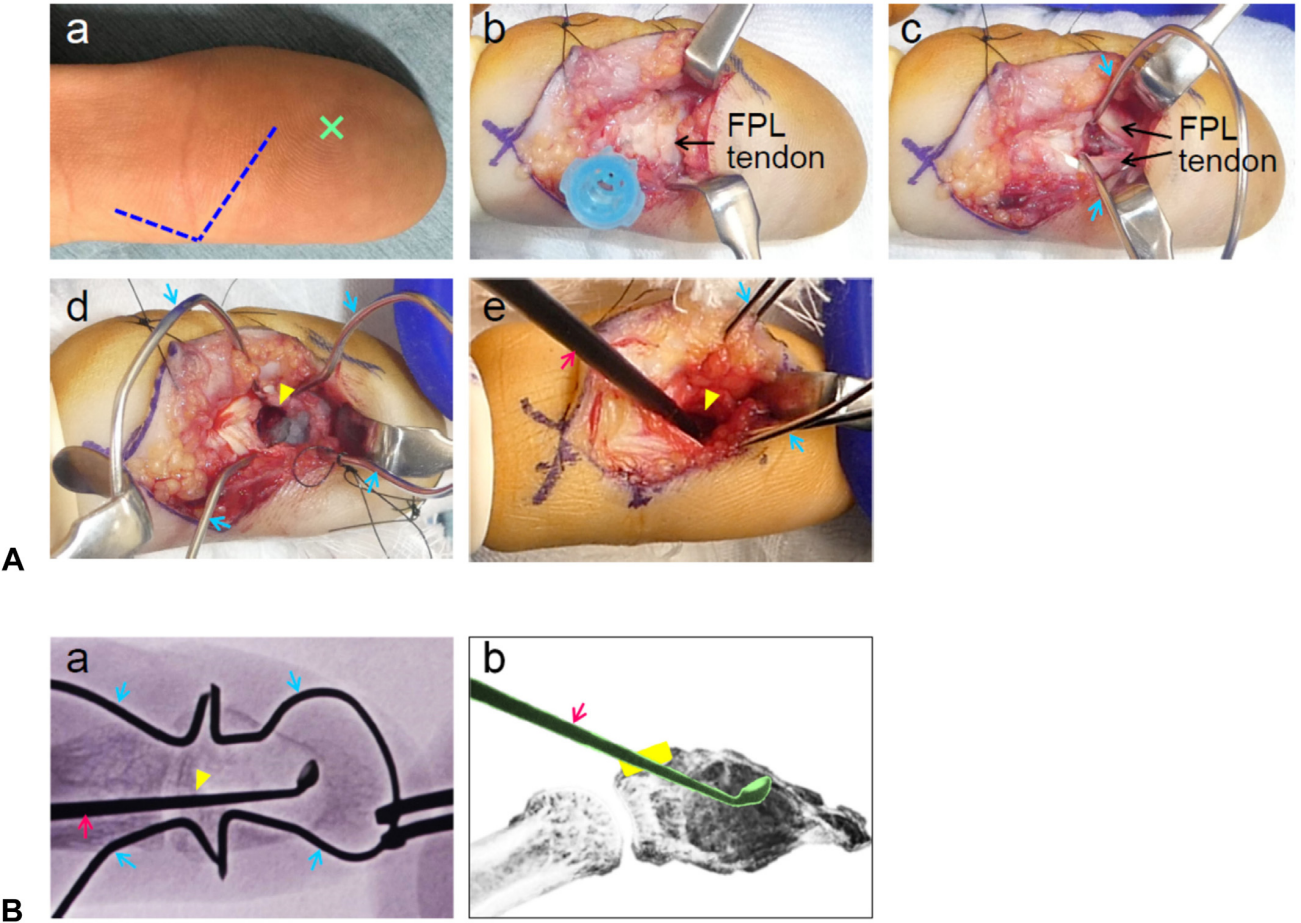
**A** Surgical procedure. (a) The incision (dotted blue line) began more than 20 mm proximal to the fingertip along the midline of the pulp. This incision extended approximately 10 mm from the proximal portion of the pulp to the outer edge of the interphalangeal crease, avoiding the central pulp (X). It was further connected to a Bruner’s incision, which extended approximately 5 mm proximally into the proximal phalanx. (b) A needle (blue cap) was inserted to confirm the level of the thumb interphalangeal joint and was used as a landmark to minimize disruption to subcutaneous tissue and fascia during dissection. (c) The insertion of the flexor pollicis longus tendon was longitudinally split, and the cortical bone was exposed using a custom-designed small retractor shaped from an omega-bent Kirschner wire (blue arrow). (d) A cortical window (yellow arrowhead, approximately 4 × 6 mm) was created using a fine scalpel blade. (e) Curettage was performed through the cortical window under fluoroscopic guidance using a small curved curette (red arrow). **B** (a) Frontal view of the fluoroscopic image during curettage. (b) Schematic illustration of curettage. Curettage was carefully performed through the cortical window (yellow) to avoid damaging the surrounding thinned cortical bone.

A 35-year-old man presented with pain in the right thumb during pinching activities. He exhibited no erythema, swelling, or range of motion limitations. Radiographs and computed tomography scans revealed a cystic lesion extending from the metaphysis to the diaphysis within the right distal phalanx (Fig. 1A, left of the blue line). No pathological fractures were present. Magnetic resonance imaging showed low signals on T1-weighted images and high signals on T2-weighted images, consistent with enchondroma. Surgery was performed using a palmar approach with Bruner’s incision, avoiding the central pulp (Fig. 2A). The patient returned to their regular desk work 2 weeks after surgery. At 1 year after surgery, most of the artificial bone had been replaced with physiological bone (Fig. 1A, right of the blue line), classified as grade 1 on the Tordai scale.^8^ The range of motion of the thumb was fully preserved (Fig. 1B).

### Case 2

**Figure 3:**
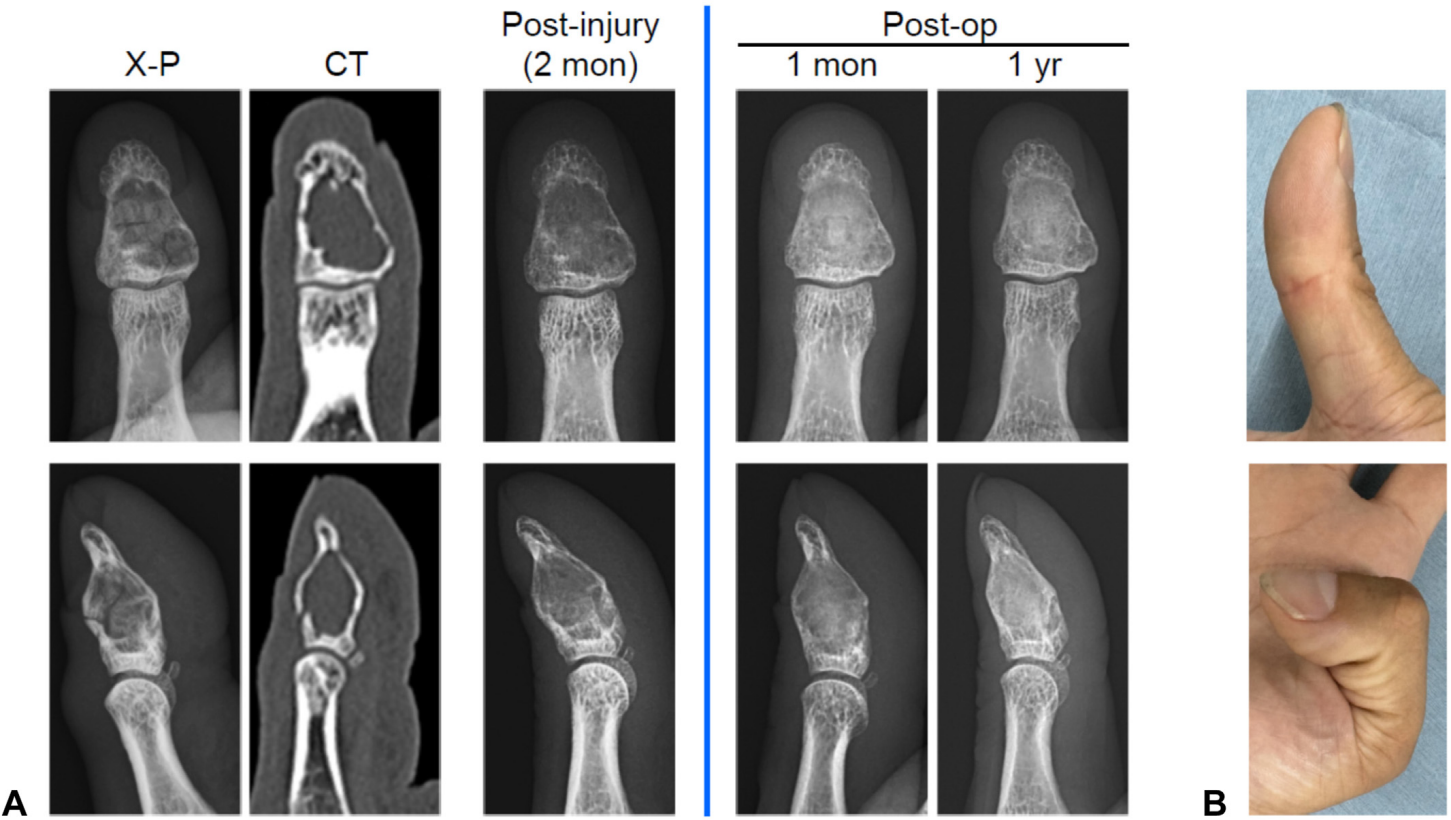
Pre- and postoperative findings for case 2. **A** Left of the blue line: initial radiographs, computed tomography, and postimmobilization radiographs at 2 months, showing frontal (upper row) and lateral (lower row) views. Right of the blue line: postoperative radiographs at 1 month and 1 year, showing frontal (upper row), and lateral (lower row) views. **B** Thumb range of motion at 1 year after surgery: extension (upper) and flexion (lower).

A 38-year-old man presented to the emergency department after experiencing severe pain in the right thumb while changing a lightbulb. Examination revealed swelling and tenderness in the thumb pad. Radiographs and computed tomography scans showed a cystic lesion extending from the metaphysis to the diaphysis with a pathological fracture of the cortical bone in the right distal phalanx (Fig. 3A, left of the blue line). After 2 months of external fixation, confirmed bone healing allowed surgery through the modified palmar approach, similar to that used in case 1 (Fig. 2). At 1 year after surgery, most of the artificial bone had been replaced with physiological bone (Fig. 3A, right of the blue line), also classified as grade 1 on the Tordai scale.^8^ The thumb’s range of motion remained fully preserved (Fig. 3B).

Histopathological examination of resected specimens confirmed immature cartilaginous tissue consistent with enchondroma. At the final follow-up, both patients had no pain, limitations in daily activities, or recurrence. Their thumb function was comparable with that of the unaffected side.

## Discussion

Our study demonstrates that the modified palmar approach using Bruner’s incision, combined with splitting the flexor pollicis longus tendon, is a feasible, and effective method for treating distal phalanx enchondromas in the thumb. This technique addresses several key limitations of traditional approaches, such as the risk of postoperative nail deformities, limited joint mobility, and painful scarring, while preserving thumb function.

Traditional skin incisions for the palmar approach to distal phalanx enchondromas typically involve a longitudinal incision extending just distal to the distal finger crease and including the central pulp.^6^^,^^7^ Although this technique prioritizes preserving range of motion by avoiding incisions across the distal finger crease, unique considerations are necessary for the thumb because of its frequent and high-pressure involvement in pinching activities. Traditional palmar incisions risk painful scarring in the central pulp, which can considerably impact daily activities.^7^ By avoiding the central pulp and minimizing soft tissue disruption, our approach considerably reduces the likelihood of complications, making it particularly suitable for lesions in functionally critical areas of the thumb.

Fluoroscopic guidance played a pivotal role in achieving complete curettage without the need for extensive exposure or direct visualization. This not only minimized intraoperative risks but also preserved the structural integrity of the surrounding bone. Our approach also allows for the strategic placement of a small cortical window, which optimizes tumor removal while avoiding unnecessary weakening of the distal phalanx.

Despite these promising results, the palmar approach poses inherent risks, including potential injury to digital nerves and vessels. Careful dissection and detailed anatomical knowledge are essential to avoid sensory deficits or vascular compromise. Furthermore, the splitting of the flexor pollicis longus tendon, although necessary for adequate exposure and curettage, raises concerns about the potential long-term weakening of the tendon insertion. In our cases, no functional deficits were observed at the 1-year follow-up, but further research is needed to assess the durability of these outcomes over longer periods.

Recent literature highlights the need for individualized strategies in enchondroma management, as factors such as lesion size, location, and patient-specific characteristics influence the optimal approach.^9^ Our findings also highlight the importance of selecting cases carefully. The technique is most effective in localized lesions, as seen in the present two cases. For more advanced cases, such as combined, and polycentric lesions according to Takigawa et al’s^10^ classification, where the tumor occupies the entire bone and considerable structural damage occurs because of pathological fractures, complete curettage using this approach may be challenging because direct visualization of the entire tumor is not feasible. Our findings support the use of the palmar approach in selected cases of distal thumb enchondromas, underscoring the importance of flexible, patient-centered surgical planning to optimize clinical and functional outcomes.

The clinical outcomes in our cases—complete bone regeneration, preservation of full range of motion, and absence of recurrence at 1 year—underscore the efficacy of this approach. Nonetheless, long-term follow-up is essential to evaluate the risk of recurrence and the structural integrity of the repaired bone. Additionally, although fluoroscopic guidance proved effective in these cases, the reliance on this modality underscores the need for advanced surgical facilities and expertise, which may limit the generalizability of this technique.

Overall, this study highlights the critical importance of individualized, patient-centered surgical planning. The modified palmar approach offers a promising option for the treatment of distal phalanx enchondromas in the thumb, particularly in cases requiring the preservation of functional and aesthetic outcomes. Future studies, including larger case series and biomechanical analyses, are needed to validate these findings and explore the broader applicability of this technique.

## Conflicts of Interest

No benefits in any form have been received or will be received related directly to this article.
